# The Gut-Brain Axis in Opioid Use Disorder: Exploring the Bidirectional Influence of Opioids and the Gut Microbiome—A Comprehensive Review

**DOI:** 10.3390/life14101227

**Published:** 2024-09-25

**Authors:** Artūras Barkus, Vaida Baltrūnienė, Justė Baušienė, Tomas Baltrūnas, Lina Barkienė, Paulina Kazlauskaitė, Augustinas Baušys

**Affiliations:** 1 Department of Pathology and Forensic Medicine, Institute of Biomedical Sciences, Faculty of Medicine, Vilnius University, 03101 Vilnius, Lithuania; 2 Laboratory of Experimental Surgery and Oncology, Faculty of Medicine, Vilnius University, 03101 Vilnius, Lithuania

**Keywords:** opioids, microbiome, microbiota, addiction, opioid use disorder, gut-brain axis

## Abstract

Opioid Use Disorder is a chronic condition characterized by compulsive opioid use despite negative consequences, resulting in severe health risks such as overdose and contraction of infectious diseases. High dropout rates in opioid agonist therapy highlight the need for more effective relapse prevention strategies. Animal and clinical studies indicate that opioids influence gut microbiota, which in turn plays a critical role in addiction development and alters behavioral responses to opioids. This study provides a comprehensive review of the literature on the effects of opioids on the gut microbiome and explores the potential of microbiome manipulation as a therapeutic target in opioid addiction.

## 1. Introduction

Opioid use disorder (OUD) stands as a manageable yet chronic condition, marked by cycles of remission and recurrence. It manifests through the loss of control over opioid use, compulsive behaviors, and continued substance use despite evident harm [1]. Left unaddressed, OUD imposes significant health and economic burdens on individuals, their families, and society at large. The first-line treatment for managing OUD is opioid agonist therapy (OAT) [1,2]. These medications not only reduce cravings but also mitigate the risk of overdose and all-cause mortality [3,4,5,6]. Furthermore, OAT contributes to a reduction in infectious disease transmission, an enhancement of the overall quality of life, and an improvement in social well-being [7,8]. Despite the benefits, the median retention rate in OAT for OUD is approximately 57% at 12 months, with a further decrease over time [9]. The exact reasons and underlying pathophysiological mechanisms for treatment failure remain elusive.

It is hypothesized that gut microbiota, through the gut-brain axis, significantly impacts the pathophysiology of addictive disorders [10]. The gut-brain axis is a bidirectional communication system between the central nervous system (CNS) and enteric nervous systems (ENS) that signals through pathways that involve neural, endocrine, immune, and humoral links [11]. One critical component of this axis is opioid receptor signaling. Opioid receptors, particularly the μ, δ, and κ subtypes, are expressed both centrally in the brain and spinal cord and peripherally in the gastrointestinal tract. Most clinical effects of opioids are mediated by μ-receptors [12], which not only play a pivotal role in pain modulation and reward processing [13] but also influence gut physiology through their presence in the ENS [14]. These receptors are found in the submucosal and myenteric ganglia of the gut, as well as on immune cells, including B cells, T cells, and macrophages [15,16], suggesting their involvement in regulating intestinal inflammation and immune responses.

Research indicates that opioid signaling, especially through μ-opioid receptors (MOR), can alter the composition of the gut microbiome. In studies using μ-opioid receptor knockout (MORKO) mice, significant differences in gut microbiota beta diversity were observed compared to wild-type mice. This suggests that the absence of MOR alters baseline microbiome composition, indicating that morphine-induced microbiome changes are mediated by MOR signaling in peripheral immune cells, with implications for opioid treatment [17].

Furthermore, morphine-mediated signaling via MORs disrupts the intestinal barrier by impairing the function of tight junctions between epithelial cells. This effect is dependent on Toll-like receptor (TLR) and IL-17A signaling, with morphine exposure increasing the expression of both in the small intestine [17,18]. Such disruptions could contribute to altered gut permeability and systemic inflammation, both of which are important in OUD pathophysiology.

Thus, the interaction between opioids, their receptors, and the gut microbiota may be a critical factor influencing OAT outcomes and the development of OUD. Microbiota-derived metabolites are also known to enter the CNS and modulate epigenetic processes [19,20,21]. Despite its complex mechanistic basis, microbiome-brain interaction correlates with a range of brain disorders, including mental health conditions [11,22,23,24], as well as specific substance use disorders [25,26,27,28]. However, the present studies on the gut microbiome in OUD are highly heterogeneous in both methodology and the outcomes investigated. Thus, this review aims to summarize the current understanding of the bidirectional association between opioid use and gut microbiota and its role in the development of OUD.

## 2. Methods

### Literature Search Strategy

A comprehensive review was conducted, including detailed searching, tabular data synthesis, and narrative commentary. The literature search was conducted using the PubMed database. The last day of the search was 17 March 2024. The following Medical Subject Heading (MeSH) terms were used during the search process: (‘microbiome’ OR ‘microbiota’) AND (‘opioid’). Time restrictions for publications were not applied. The inclusion criteria were:

1. Original research articles (clinical or basic science) on opioid use, including sequencing and analysis of the gastrointestinal microbiome;
2. Studies presenting data on microbial composition, diversity, or function;
3. Peer-reviewed articles, written in English.

**Figure 1:**
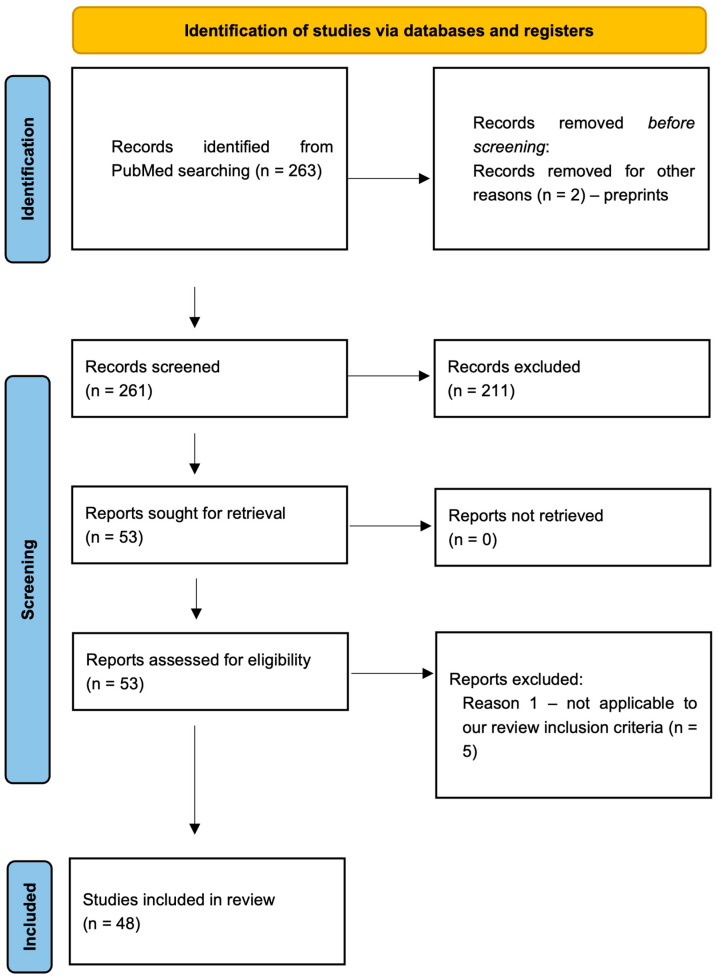
Literature search flow diagram.

The exclusion criteria were articles that do not apply to inclusion criteria, editorials, letters, conference reports, review articles, and systematic reviews. We also excluded articles that focus on peripherally acting opioid agonists without inherent potential for OUD at therapeutic doses. Initially, articles were screened based on their titles and abstracts by two independent and experienced reviewers (A. B. and V. B.). Once relevant abstracts were identified, the full-text articles were retrieved and reviewed for inclusion. Additionally, a manual search of the reference lists was performed to ensure a comprehensive literature search procedure. Then, all original clinical and experimental studies investigating the association between the gut microbiome and opioid use were included in this comprehensive review (Figure 1). Institutional review board approval was not required.

## 3. Results

**Figure 2:**
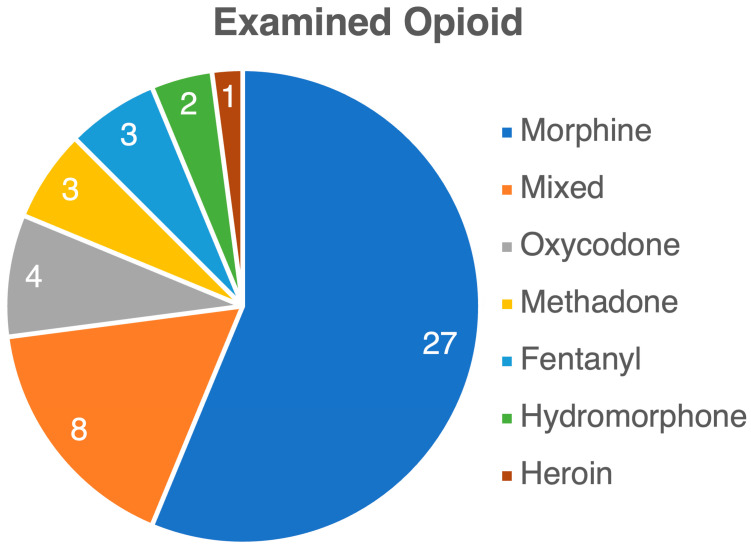
Number of reviewed studies by examined opioid.

**Figure 3:**
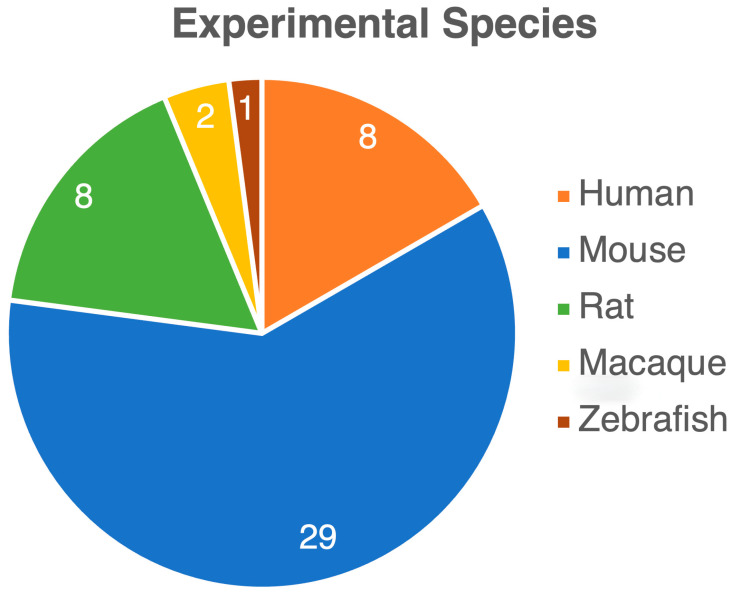
Number of reviewed studies by experimental species.

**Figure 4:**
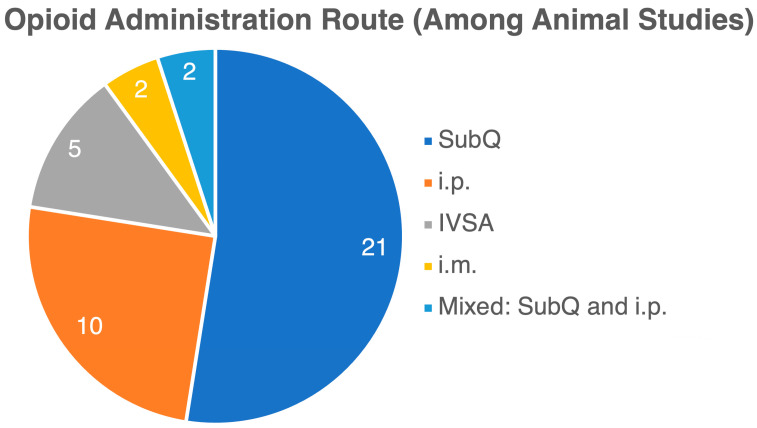
Number of reviewed animal studies by opioid administration route. Abbreviations: SubQ—subcutaneous; i.p.—intraperitoneal; IVSA—intravenous self-administration; i.m.—intramuscular.

Through a literature search, we identified 48 manuscripts, consisting of 40 experimental and 8 clinical studies, that investigated the impact of opioids on the gut microbiome. While all studies focused on opioids’ effects on the gut microbiome, there were significant variations in methodology and measured outcomes. Clinical studies varied widely in patient cohorts and comorbidities, including individuals in addiction centers and those with severe diseases like liver cirrhosis and cancer. These studies also examined different opioid types and regimens, with controls ranging from healthy individuals to non-opioid users or no controls at all. In preclinical studies, various species—including rodents, macaques, and zebrafish—were used, with diverse opioid types and regimens. The outcomes were inconsistent, with some studies reporting decreased gut microbiota biodiversity, others showing increases, and some no change at all. One Mendelian randomization study found no clear link between opioid use and microbiota changes. Methodologies also differed, with clinical studies being cross-sectional and experimental studies using longitudinal designs, applying either 16S rRNA sequencing or metagenomic approaches. Table 1 summarizes the included studies. Figure 2, Figure 3 and Figure 4 present the variety of studies by the examined opioid, experimental species, and route of opioid administration in animal studies, respectively.

### 3.1. Opioid Use Impact on Gut Microbiome: Changes in Gut Microbiome Composition and Diversity

Current evidence from clinical and experimental studies indicates that opioid use is associated with specific changes in the gut microbiome, commonly referred to as opioid-induced dysbiosis [29]. Opioid use impact on various parameters describing gut microbiome composition is detailed below.

#### 3.1.1. Opioid Use Impact on α-Diversity and β-Diversity

Numerous studies, both basic science [17,30,31,32,33,34,35,36,37,38,39,40,41,42,43,44,45,46,47,48,49,50,51,52,53,54,55] and clinical [56,57,58,59,60], have investigated the impact of opioid use on gut microbial alpha diversity. Most employed standard metrics such as the Chao1 index, Shannon index, Simpson index, and Operational Taxonomic Units (OTUs), collectively providing insights into the richness, evenness, and overall diversity of gut microbial communities [31,32,33,34,35,36,37,38,39,40,41,42,44,45,46,47,48,49,50,51,52,53,54,55,56,57,58,59,60]. However, the current evidence remains controversial. On the one hand, several experimental studies have reported no significant impact of opioids, including morphine, oxycodone, and heroin, on the alpha diversity of the gut microbiome [17,37,38,43,48,49,51,54,55,61]. Similarly, a study on maternal hydromorphone exposure showed no impact on alpha diversity in dams or their offspring [31]. These findings have been corroborated in clinical settings, including the study by Xu et al., which found no significant differences in alpha diversity between individuals with substance use disorders, predominantly comprising heroin users, and healthy controls [60]. Li et al. also reported no significant differences in alpha diversity among participants receiving methadone maintenance treatment, current drug users, healthy controls, and individuals in compulsory detention [58]. Additionally, Wang et al. observed no significant differences in alpha diversity between control and opioid-exposed groups in patients with cancer pain treated with oxycodone [59]. On the other hand, contrary to these findings, some studies indicate that opioids have an impact on alpha diversity, although the nature of this impact is ambiguous. Morphine and hydromorphone have been shown to decrease alpha diversity in mouse models [35,45,47,50], with similar effects observed in morphine-treated zebrafish and macaques [34,40]. Additionally, opioid agonist-induced reductions in alpha diversity have been reported in clinical studies [56,57]. Conversely, Jalodia et al. reported a significant increase in gut microbiome alpha diversity following morphine treatment in mice [39]. Likewise, repeated morphine administration in mice led to higher gut microbial community richness during the acquisition of morphine-induced conditioned place preference (CPP) [52]. Kesh et al. found that mice with chronic pancreatitis (CP), mice treated with morphine, and mice with CP treated with morphine (CP + morphine) all showed higher alpha diversity compared to healthy controls [41]. This pattern of increased alpha diversity was also consistent in oxycodone-treated CP animals in their study. Moreover, Grecco et al. also reported elevated levels of both evenness and richness in the gut microbiota of methadone-treated dams and their offspring in mice [36]. A potential explanation for increased alpha diversity could be attributed to a persistent decrease in gastrointestinal motility caused by opioids [62]. Few studies have shown the influence of age and sex on how opioids affect alpha diversity. Antoine et al. found that neonatal mice exposed to morphine initially exhibited higher alpha diversity compared to saline-exposed mice, but no significant differences were observed by adulthood [32]. Ren and Lotfipour demonstrated that both sex and dose determine the impact of intravenous fentanyl self-administration (IVSA) in rats on the alpha diversity of gut bacteria, with fentanyl IVSA increasing diversity in males at 1.25 μg/kg/infusion and decreasing alpha diversity in females at the same dose [46].

Beta diversity was another commonly investigated parameter describing the gut microbiome, which was analyzed using UniFrac distances and Bray–Curtis indices. Similar to the findings for alpha diversity, the results regarding the impact of opioids on beta diversity have shown contradictory results; some studies reported no effect on beta diversity [38,44,46,54,57,59,61], while others demonstrated notable changes associated with opioid use [17,30,31,32,34,36,37,39,41,42,43,45,47,49,50,52,53,55,56,58,60,63,64,65].

Experimental studies in rodents [17,30,31,37,41,42,43,45,47,50,52,64,65], zebrafish, and macaques [34,49] have shown that opioids, including morphine and heroin, significantly alter beta diversity, leading to distinct clustering of microbial communities compared to control groups. Furthermore, the impact of opioids on beta diversity has been confirmed in human studies as well [56,58,60,63].

Overall, the effects of opioids on both alpha and beta diversity vary significantly across studies and experimental conditions. While some research indicates notable reductions in alpha diversity, others observe no significant changes or even increases. Similarly, studies diverge on the effects of opioids on beta diversity.

#### 3.1.2. Opioid Use Impact on Microbial Taxonomic Composition

Most studies evaluating the influence of opioids on gut microbiome composition have revealed distinct dysbiotic changes [17,30,31,32,33,34,35,36,37,38,39,40,41,42,43,44,45,46,47,48,49,50,51,52,55,56,57,58,59,60,63,64,65,66,67,68]. The gut microbiome is predominantly composed of *Firmicutes* (including genera *Clostridium*, *Enterococcus*, *Lactobacillus*, and *Ruminococcus*) and *Bacteroidetes* (including genera *Bacteroides* and *Prevotella*) phyla [69], with the Firmicutes/Bacteroidetes (F/B) ratio commonly evaluated as a measure of dysbiosis. Previous studies have linked alterations in these phyla with inflammation and various diseases, including diabetes, obesity, and inflammatory bowel disease [70,71,72,73,74,75]. However, findings on its relation to diseases, e.g., obesity [76], appeared mixed, and age-related variability was also observed [77,78]. Additionally, studies on opioids have also yielded heterogeneous results regarding F/B ratio, making its interpretation challenging. For instance, some studies exploring morphine’s effect in mice reported an increase in the Firmicutes/Bacteroidetes ratio [17,32,45,53], whereas others investigating the morphine effect in rodents and other animals reported a decrease [34,39,40,68]. Additionally, some studies have only reported an increase in *Firmicutes* [65], while others have noted a decrease in *Bacteroidetes* [48,67].

**Figure 5:**
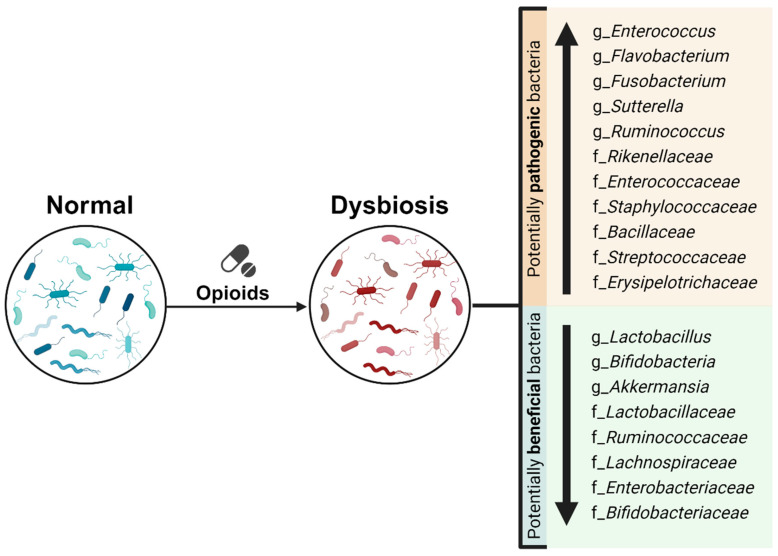
Characteristics of opioids induced dysbiosis. Current studies show that opioid use is associated with gut dysbiosis, characterized by the expansion of potentially pathogenic bacteria and a decrease in potentially beneficial bacteria. Figure was created with BioRender.com.

Opioid use has been associated with the expansion of potentially pathogenic bacteria, including genera like *Enterococcus*, *Flavobacterium*, *Fusobacterium*, *Sutterella*, *Clostridium*, and *Ruminococcus* [37,39,42,43,45,47,50,53,58,64,66], and families such as *Rikenellaceae*, *Enterococcaceae*, *Staphylococcaceae*, *Bacillaceae*, *Streptococcaceae*, and *Erysipelotrichaceae* [17,39,43,53,64]. However, some studies have reported a decrease in some of these taxa (*Ruminococcus*, *Enterococcaceae*, *Clostridiales*, *Clostridium*) in relation to opioid use [31,51,58,67]. Not all species within these genera are inherently pathogenic. For instance, *Clostridia* are abundant in the healthy distal ileum and colon. Commensal *Clostridia* are known for their anti-inflammatory effects through fiber metabolism, SCFA production (especially butyrate), and the induction of regulatory T cells that produce TGF-β and IL-10 [79,80,81]. Similarly, studies on opioid use have reported a decrease in potentially beneficial bacteria, such as *Lactobacillus* and *Bifidobacteria* [31,39,41,42,43,45,47,53,65], and other bacteria from the families *Lactobacillaceae*, *Ruminococcaceae*, *Lachnospiraceae*, *Enterobacteriaceae*, and *Bifidobacteriaceae*, which are associated with SCFA production [17,35,36,37,40,45,47,56,57,63]. In a study on methadone effects in patients with OUD, Cruz-Lebrón et al. noted a decrease in a specific species, *Akkermansia muciniphila* [56], associated with several beneficial effects, including the strengthening of intestinal epithelial integrity [82,83,84]. However, some studies have found an increase in *Lactobacillus*, *Bifidobacteria*, and the *Akkermansia* genera in relation to opioid use [31,36,47,56,57,58,59,66] (Figure 5).

The reviewed studies consistently suggest that opioid use correlates with gut dysbiosis. However, characterizing this microbial shift solely as pathogenic presents challenges due to mixed findings across specific bacterial taxa. While some studies report an increase in potentially pathogenic bacteria, others identify a decrease in beneficial bacteria.

The impact of opioids on gut microbiome composition, including alpha and beta diversity, appears highly heterogeneous across studies. These discrepancies may arise from diverse factors, including study populations (zebrafish, rats, mice, macaques, and humans), study methodology, sample sizes, differences in study parameters like gender, and the specific types and administration regimens of opioids used. Individual differences in baseline microbiome, diet, health status, and environment also contribute to the variability.

Interestingly, a two-sample bidirectional Mendelian randomization study found potential causal effects of seven genetically influenced gut microbiome traits on prescription opioid use but did not find clear evidence for a significant causal relationship in the reverse direction—the influence of prescription opioid use on changes in the gut microbiome [85]. These varying outcomes underscore the complexity of the gut microbiome and the need for standardized methods and larger, comprehensive studies to clarify opioid effects on gut microbiome.

**Table 1:** Studies included in this review investigating the impact of opioids on gastrointestinal microbiome.

No.	Author	Experimental Species	Examined Opioid	Treatment Protocol	Opioids Impact on Gut Microbiome Diversity	Opioids Impact on Gut Microbiome Composition	
						Increased Abundance	Decreased Abundance
1.	Meng et al., 2013 [18]	Mouse	Morphine	75 mg pellet for 24 h SubQ	No data	No data	No data
2.	Meng et al., 2015 [64]	Mouse	Morphine	25 mg pellet for 3 days SubQ	**Alpha diversity:** no data **Beta diversity:** Morphine-treated CLP animals clustered distinctly from the Placebo-treated and Placebo-treated CLP animals	Genera: *Staphylococcus*; *Enterococcus*. *Species:* *Staphylococcus sciuri*, *Staphylococcus cohnii*, *Staphylococcus aureus*, *Enterococcus durans*, *Enterococcus casseliflavus*, *Enterococcus faecium* and *Enterococcus faecalis*	No data
3.	Banerjee et al., 2016 [17]	Mouse	Morphine	25 mg pellet for 2 days SubQ	**Alpha diversity:** no significant changes **Beta diversity:** altered composition and distinct clustering between study groups	↑ Diversity of *Firmicutes* Families (from phylum *Firmicutes*): *Enterococcaceae*, *Staphylococcaceae*, *Bacillaceae*, *Streptococcaceae*, *Erysipelotrichaceae*	↓ Bile-deconjugating bacterial strains Phylum: *Bacteroidetes*; Reduced Bacteroidetes/Firmicutes ratio
4.	Acharya et al., 2017 [63]	Human	Mixed: Oxycodone, morphine, hydromorphone, tramadol, methadone	Opioid-using patients were on therapy for a median of 5 months	**Alpha diversity:** no data **Beta diversity:** altered composition and distinct clustering between study groups (HE patients on opioids compared to HE patients not on opioids)	HE: *Bifidobacterium* (*genus*)	HE: *Bacteroidaceae* and Autochthonous taxa (*Clostridiales XI* (family), *Ruminococcaceae* (family))
						Non-HE: *Peptostreptococcaceae* (family)	Non-HE: *Parasutterella* (*genus*)
5.	Kang et al., 2017 [67]	Mouse	Morphine	75 mg pellet for 5 days SubQ	No data	*Phylum:* *Proteobacteria* (*Enterobacteriales*)	Phyla: *Bacteroidetes* (*Bacteroidales*) *Firmicutes* (*Clostridiales*, *Lactobacilliales*)
6.	Xu et al., 2017 [60]	Human	Mixed: heroin, ice, ephedrine, heroin + ephedrine, and heroin + ice	-	**Alpha diversity:** higher in SUDs compared to the HCs, but no significant differences between the groups **Beta diversity:** altered composition and distinct clustering between study groups (SUDs vs. HCs)	Genera: *Prevotella*, *Ruminococcus*, *Phascolarctobacterium*, *Alloprevotella*, *Megamonas*, *Roseburia*, *Clostridium XlVa*	Genera: *Bacteroides*, *Faecalibacterium*, *Alistipes*, *Gemmiger*, *Clostridium XI*, *Escherichia*/*Shigella*, *Dialister*, *Paraprevotella*, *Megasphaera*, *Haemophilus*, *Parabacteroides*, *Barnesiella*, *Blautia*
7.	Barengolts et al., 2018 [66]	Human	Mixed	The DSM-4 diagnostic criteria for Opioid Use Disorder	No data	Phylum: *Actinobacteria*, Order: *Lactobacillales*, *Bifidobacteriales* Genus: *Bifidobacterium*	Species: *Prevotella copri*
8.	Lee et al., 2018 [43]	Mouse	Morphine	Intermittent: 10, 20, 30, 40 mg/kg every 12 h i.p. for 4 days	**Alpha diversity:** no significant changes **Beta diversity:** altered composition and distinct clustering between study groups (intermittent or sustained morphine vs. controls)	↑ *Ruminococcus* spp.	↓ *Lactobacillus* spp.
				25 mg pellet for 4 days SubQ		↑ *Clostridium* spp. ↑ *Rikenellaceae* spp.	-
9.	Mischel et al., 2018 [86]	Mouse	Morphine	75 mg pellet SubQ	No data	No data	No data
10.	Wang et al., 2018 [50]	Mouse	Morphine	25 mg pellet for 3 days SubQ	**Alpha diversity:** reduced **Beta diversity:** altered composition and distinct clustering between study groups	Pathogenic genera: *Flavobacterium*, *Enterococcus*, *Fusobacterium*, *Sutterella*, *Clostridium* *Enterococcus faecalis* (species)	-
11.	Hakimian et al., 2019 [87]	Mouse	Remifentanil, oxycodone	IVSA: 3 days 0.05 mg/kg/infusion of remifentanil; 10 days 0.25 mg/kg/infusion of oxycodone	No data on opioid vs. control groups	No data on opioid vs. control groups	No data on opioid vs. control groups
12.	Komla et al., 2019 [88]	Mouse	Morphine	25-, 50- (2 × 25), or 75-mg pellets for 3–5 days SubQ	No data	No data	No data
13.	Meng et al., 2019 [45]	Mouse	Morphine	75 mg pellet for 7 days SubQ	**Alpha diversity:** reduced **Beta diversity:** altered composition and distinct clustering between study groups	HIV + Morphine: Phyla: *Firmicutes*, *Proteobacteria* Genus: *Enterococcus*	HIV + Morphine: Phyla: *Bacteroidetes*, *Actinobacteria*, and *Tenericutes*; Families: *Muribaculaceae*, *Lachnospiraceae*, and *Ruminococcaceae* Genus: *Lactobacillus*
14.	O’Sullivan et al., 2019 [68]	Rat	Morphine (withdrawal)	2 × 75 mg pellets for 6 days SubQ	No data	Placebo vs. morphine groups—no statistical difference.	Placebo vs. morphine groups—no statistical difference.
						Withdrawal: Phyla: *Bacteroidetes*, *Verrucomicrobia* Species: *Bacteroides fragilis*, *B. vulgatus* and *B. thetaiotaomicron*, *Enterococcus faecalis*, *Enterococcus gallinarum*	Withdrawal: Phyla: *Firmicutes*, *Actinobacteria* Genera: *Butyricicoccus*, *Bifidobacterium* Species: *Butyricicoccus pullicaecorum*, *F. prausnitzii* ↓ *Firmicutes* to *Bacteroides* ratio
15.	Sindberg et al., 2019 [49]	Macaque	Morphine	2à4 mg/kg every 8 h i.m. for 12 weeks	**Alpha diversity:** no significant changes **Beta diversity:** altered composition and distinct clustering between pre and post morphine induction	-	Family: *Leuconostocaceae* Genera: *Streptococcaceae streptococcus*, *Pasteurellaceae Aggregatibacter*
16.	Zhang et al., 2019 [65]	Mouse	Morphine	8 days: constant dose of 15 mg/kg or escalating doses of (5, 10, 15, 20, 25, 30, 35, 40 mg/kg) morphine injection b.i.d intraperitoneally	**Alpha diversity:** no data **Beta diversity:** altered composition and distinct clustering between study groups (morphine-tolerant vs. saline-treated mice); no difference in TLR2KO and TLR4KO mice	Phyla: *Actinobacteria* and *Firmicutes*	Families: *Bifidobacteriaceae* and *Lactobacillaceae*; Genera: *Bifidobacterium* and *Lactobacillus*
17.	Chen et al., 2020 [34]	Zebrafish	Morphine	On days 4, 6, and 8 injected with morphine (40 mg/kg)	**Alpha diversity:** decreased **Beta diversity:** altered composition and distinct clustering between study groups	Phylum: *Fusobacteria* ↑ *Bacteroidetes*/*Firmicutes* (B/F) ratio	Phylum: *Actinobacteria*
18.	Gicquelais et al., 2020 [57]	Human	Mixed: Ag only (heroin or PO), AgAt (buprenorphine–naloxone and PO and naltrexone), At (naltrexone only), N (neither opioid agonist nor antagonist)	participants from the patient population attending a private, outpatient addiction treatment facility in Michigan	**Alpha diversity:** decreased (Ag vs. N) No significant changes (AgAt and At vs. N) **Beta diversity:** no distinct clustering between study groups	Ag vs. N: Genera: Unclassified Enterobacteriaceae, *Lactobacillus*, *Clostridium* cluster XIVa, *Faecalicoccus*, *Anaerostipes*, and *Streptococcus* No statistically significant differences between AgAt vs. N or At vs. N participants.	Ag vs. N: Genera: Unclassified Firmicutes, *Bilophila*, and *Roseburia*
19.	Li et al., 2020 [58]	Human	Methadone; illicit drugs	MMT patients; current drug using (DU) participants with narcotic (heroin) and psychotropic (methamphetamine) drug use disorders; healthy controls; compulsory detention (CD)	**Alpha diversity:** no significant changes **Beta diversity:** significantly higher among MMT patients	**MMT patients:** Phyla: Cyanobacteria chloroplast and Actinobacteria. Genera: *Lactobacillus*, *Streptococcus*, *Veillonella*, *Bifidobacterium*, *Intestinibacter*, *Fusicatenibacter*; *Streptococcus* (*genus*) and *Fusicatenibacter* (*genus*) (MMT vs. CD and DU); *Klebsiella* (MMT vs. CD).	-
						**DU:** Genera: *Ruminococcus*, *Roseburia*, *Collinsella*, and *Succinivibrio*	
20.	Sharma et al., 2020 [47]	Mouse	Hydromorphone	7.5 mg/kg every 12 h × 7 d i.p.	**Alpha diversity:** decreased **Beta diversity:** altered composition and distinct clustering between study groups	Hydromorphone plus DSS-treated mice compared with control mice: Phyla: *Proteobacteria*, *Verrucomicrobia* Families: *Bacteroidaceae*, *Porphyromonadaceae*, *Enterococcaceae*, *Enterobacteriaceae*, *Verrucomicrobiaceae*, and *Peptostreptococcaceae* Genera: *Bacteroides*, *Parabacteroides*, *Enterococcus*, *Turicibacter*, *Ruminococcus*, *Sutterella*, *Bilophila*, and *Akkermansia* Species: *Bacteroides acidifaciens*, *Ruminococcus gnavus*, and *Akkermansia muciniphila*	Hydromorphone plus DSS-treated mice compared with control mice: Phylum: *Firmicutes* Families: *Odoribacteraceae*, *Rikenellaceae*, *S24-7*, *Lactobacillaceae*, *Lachnospiraceae*, and *Ruminococcaceae* Genera: *Adlercreutzia*, *Odoribacter*, *AF12*, *Lactobacillus*, and *Anaerostipes* Species: *Mucispirillum schaedleri* and *Lactobacillus reuteri*
21.	Simpson et al., 2020 [48]	Rat	Oxycodone	2 mg/kg every 12 h for 5 days SubQ	**Alpha diversity:** no significant changes **Beta diversity:** no data	*Firmicutes* (phylum)	*Bacteroidetes* (phylum)
22.	Zhang et al., 2020 [51]	Rat	Morphine	10 mg/kg i.p. (day 6, 8, 10, 12)	**Alpha diversity:** no significant changes **Beta diversity:** no data	M-Post-treatment vs. M-Baseline: Genera: *Allobaculum*, *Parasutterella* Families: *Coriobacteriaceae* and *Peptococcaceae_1*	M-P vs. M-B: Genera: *Alloprevotella*, *Desulfovibrio*, *Rikenella*
						MP vs. SalineP: -	MP vs. SalineP: Genera: *Corynebacterium*, and *Clostridium_XlVa* Families: *Enterococcaceae*, *Staphylococcaceae*, *Streptococcaceae*
23.	Cruz-Lebrón et al., 2021 [56]	Human	Methadone	Methadone-treated individals	**Alpha diversity:** decreased **Beta diversity:** significantly decreased	Phylum: *Actinobacteria*; Family: *Bifidobacteriaceae*; Species: *Bifidobacterium bifidum* and *Bifidobacterium longum*	Phylum: *VerrucomicrobiaI*; Family: *Akkermasiaceae*; Species *Akkermansia muciniphila*
24.	Grecco et al., 2021 [36]	Mouse	Methadone, oxycodone	Oxycodone 10 → 30 mg/kg twice daily for 9 days SubQ → methadone 10 mg/kg (mothers). These treatments continued throughout mating, pregnancy, and weaning (60 days)	**Alpha diversity:** increased in methadone-treated dams and PME offspring **Beta diversity:** distinct clustering by treatment for dams, but this clustering was lost in offspring	Dams: Famillies: *Erysipelotrichaceae*, *Peptostreptococcaceae*, *Akkermansiaceae*, *Lactobacillaceae*, *Sutterellaceae*, *Eubacterium Coprostanoligenes Group*, *Anaerovoracaceae*, *Monoglobaceae*, *Eggerthellaceae*	-
						In offspring: Families: *Rikenellaceae*, *Peptococcaceae*, *Saccharimonadaceae*	
						In both **dams** and **offspring**: Families: *Ruminococcaceae*, *Rikenellaceae*, *Bacteroidaceae*, *Erysipelotrichaceae*, *Acholeplasmataceae*, *Peptococcaceae*	
25.	Hofford et al., 2021 [38]	Mouse	Morphine	10 mL/kg twice daily for 7 days SubQ	**Alpha diversity:** no significant changes **Beta diversity:** near complete overlap between samples in H_2_O-Sal and H_2_O-Mor groups and relative proportions of bacterial phyla were similar between H_2_O-Sal and H_2_O-Mor	Morphine itself had minimal effects on microbiome composition	Phyla: *Rokubacteria* and *Cyanobacteria* However, these phyla are expressed at very low abundance in all tested groups (both <0.1%).
26.	Thomaz et al., 2021 [54]	Mouse	Morphine	Twice daily for 2 days (day 1: 7.5 and 15 mg/kg; day 2: 30 and 30 mg/kg) i.p.	**Alpha diversity:** no significant changes **Beta diversity:** no distinct clustering between study groups	Phylum: *Verrucomicrobia*	Phylum: *Firmicutes*
27.	Zhang et al., 2021 [52]	Mouse	Morphine	10 mL/kg twice daily i.p. (on days 3, 5, 7, 9, 11 and 13)	**Alpha diversity:** increased richness but not diversity **Beta diversity:** altered composition and distinct clustering between study groups (different stages of morphine-induced CPP (acquisition, extinction, and reinstatement) and controls)	Phylum: *Verrucomicrobia*	
						Acquisition stage: -	Acquisition stage: Genus: *Bacteroides*
						Extinction stage: Genera: *Bacteroides* and *Coprobacter*	Extinction stage: Genera: *Akkermansia*, *Saccharibacteria_genera_incertae_sedis*, *Eisenbergiella*, and *Ruminococcus*
						Phyla: *Verrucomicrobia* abundance increased in the acquisition of morphine CPP group compared to the control and decreased at the extinction stage compared to the acquisition stage, indicating an expansion response of Verrucomicrobia to morphine treatment.	*Bacteroides* was the genus that decreased after repeated morphine conditioning and had a recovery trend at the extinction stage.
28.	Abu et al., 2022 [30]	Mouse	Hydromorphone	10 mg/kg once daily i.p. Gestational day 11–13 (prenatal opioid exposure)	Four days post last hydromorphone (Dams): **Alpha diversity:** no significant changes	Four days post last hydromorphone (Dams): Phyla: *Bacteoidetes* and *Proteobacteria*; Genera: *Bacteroides* (from phylum *Bacteoidetes*) and *Sutterella* (from phylum *Proteobacteria*)	Four days post last hydromorphone (Dams): Phylum *Firmicutes*; Genera *Adlercreutzia* (from phylum *Actinobacteria*), and *Anaerostipes* (from phylum *Firmicutes*)
					Birth: **Alpha diversity:** no significant changes **Beta diversity:** microbial composition of fecal samples from Prenatal opioid exposure (POE) mothers was significantly different from control mothers 4 dp last hydromorphone/saline treatment and at parturition	Birth: Phyla: *Bacteoidetes* and *Proteobacteria*; Genera: *Bacteroides* (from phylum *Bacteoidetes*) and *Turicibacter* (from phylum *Firmicutes*)	Birth: Phylum *Firmicutes*; Genera: *Allobaculum* (from phylum *Firmicutes*) and *Roseburia* (from phylum *Firmicutes*)
					Weaning: **Alpha diversity:** no significant changes	Weaning: Phylum *Verrucomicrobia*; Genera: *Clostridium* (from phylum *Firmicutes*) and *Akkermansia* (from phylum *Verrucomicrobia*)	Weaning: Phylum *Firmicutes*; Genera: *Oscillospira* (from phylum *Firmicutes*) and *Roseburia* (from phylum *Firmicutes*)
					2 weeks: **Alpha diversity:** no significant changes **Beta diversity:** the microbial composition in POE offspring was significantly different from controls	2 weeks: Phylum *Firmicutes*; Genera: *Lactobacillus*, *Ruminococcus* and *Allobaculum* (all genera from phylum *Firmicutes*)	2 weeks: Phyla: *Verrucomicrobia* and *Tenericutes*; Genera: *Akkermansia* (from phylum *Verrucomicrobia*), *Clostridium* (from phylum *Firmicutes*), and *Bifidobacterium* (from phylum *Actinobacteria*)
					3 weeks: **Alpha diversity:** no significant changes **Beta diversity:** the microbial composition in POE offspring was significantly different from controls	3 weeks: Genera: *Turicibacter* (from phylum *Firmicutes*), *Bacteroides* (from phylum *Bacteoidetes*), *Bifidobacterium* (from phylum *Actinobacteria*), *Allobaculum* (from phylum *Firmicutes*) and *Dehalobacterium* (from phylum *Firmicutes*)	3 weeks: Phyla: *Verrucomicrobia* and *Proteobacteria*; Genera: *Akkermansia* (from phylum *Verrucomicrobia*), *Coprobacillus* (from phylum *Firmicutes*), *Dorea* (from phylum *Firmicutes*) and *Adlercreutzia* (from phylum *Actinobacteria*)
					5 weeks: **Alpha diversity:** increased **Beta diversity:** no significant difference	5 weeks: Genera: *Ruminococcus* (from phylum *Firmicutes*)	5 weeks: Genera: *Lactobacillus* (from phylum *Firmicutes*) and *Staphylococcus* (from phylum *Firmicutes*)
					**Alpha diversity:** no significant changes (stomach) **Beta diversity:** stomach samples from POE mice were significantly different from control mice	Offspring stomach: Genera: *Staphylococcus* (from phylum *Firmicutes*) and *Lactobacillus* (from phylum *Firmicutes*)	Offspring stomach: Genera: *Akkermansia* (from phylum *Verrucomicrobia*), *Clostridium* (from phylum *Firmicutes*) and an unknown genus from family S24-7 (from phylum *Bacteoidetes*)
29.	Antoine et al., 2022 [32]	Mouse	Morphine	post-natal day 7 ± 2 days for a duration of 7 ± 2 days total: 5 mg/kg/day once-a-day SubQ	Adolescence: **Alpha diversity:** increased **Beta diversity:** no significant difference	Adolescence: Phylum Firmicutes Increase in the *Firmicutes*/*Bacteroidetes* (F/B) ratio (female only);	Adolescence: Phyla: *Bacteroidetes*, *Verrucomicrobia* and *Actinobacteria* (female only); Genera: *Lactobacillus* (phylum *Firmicutes*), *Turicibacter* (phylum *Firmicutes*), *Akkermansia* (phylum *Verrucomicrobiota*) and *Bifidobacterium* (phylum *Actinobacteria*)
					Adulthood: **Alpha diversity:** no significant changes **Beta diversity:** altered composition and distinct clustering between study groups	Adulthood: Phylum *Firmicutes* (both male and female). The increase in the F/B ratio appeared later in life for male mice Genera: *Allobaculum*, *Lactobacillus* and *Turicibacter*	Adulthood: Phyla: *Bacteroidetes* and *Verrucomicrobia* Genus *Akkermansia*
30.	Blakeley-Ruiz et al., 2022 [33]	Mouse	Morphine	Subcutaneous osmotic pump: 10 mg/kg/day for 2 weeks	**Alpha diversity:** no significant changes **Beta diversity:** no data	-	Metaproteomic analysis: Proteins from species: *Eubacterium* sp. or *Lachnospiraceae bacterium*
31.	Jalodia et al., 2022 [39]	Mouse	Morphine	25 mg pellet for 24 h SubQ	**Alpha diversity:** increased **Beta diversity:** altered composition and distinct clustering between study groups	Genera: *Staphylococcus*, *Enterococcus*, and *Bacteroides*;	Reduced *Firmicutes* to *Bacteroidetes* ratio *Lactobacillus* genus
32.	Ji et al., 2022 [55]	Mouse	Morphine	10 mg/kg, once a day for 6 days i.p.	**Alpha diversity:** no significant changes **Beta diversity:** distinct clustering between study groups	Mor-dep vs. control: Genera: *Coprobacter* and *Enterorhabdus*	Mor-Dep vs. control: Genus: *Anaerotruncus*
						Mor-nondep vs. control: Genus: *Coprobacter*	Mor-nondep vs. control: Genera: *Eisenbergiella* and *Anaerotruncus*
33.	Johnson et al., 2022 [40]	Macaque	Morphine	Increased dose within 2 weeks to 6 mg/kg twice a day (i.m.) à 7 more weeks à infection with SIV	**Alpha diversity:** decreased **Beta diversity:** no data	Phylum *Bacteroidetes*; Family *Prevotellaceae*	Phylum *Firmicutes*; Family *Ruminococcaceae*
34.	Lin et al., 2022 [85]	Human	Prescription opioids (e. g. morphine, oxycodone, codeine, fentanyl, pethidine, and tramadol)	A two-sample bi-directional Mendelian randomization using summary level Genome-wide association studies	No data	no clear evidence for any causal effect of POU on gut microbiota	no clear evidence for any causal effect of POU on gut microbiota
35.	Lyu et al., 2022 [44]	Mouse	Oxycodone	5 mg/kg for 2 weeks i.p. prior to breeding and then throughout gestation	Female offspring (adulthood): **Alpha diversity:** no significant changes **Beta diversity:** no significant difference	Female offspring (adulthood): Phylum: *Bacteroidetes*, TM7 Class: *Clostridia* Genera: *Butyricimonas* spp., *Anaeroplasma* spp., *Enterococcus* spp.	Female offspring (adulthood): Genus: *Clostridium* spp.
					Male offspring (adulthood): **Alpha diversity:** no significant changes **Beta diversity:** no significant difference	Male offspring (adulthood): Family: *Coriobacteriaceae* Class: *Clostridia* Genera: *Roseburia* spp., *Sutterella* spp.	Male offspring (adulthood): Phylum: *Firmicutes* Class: *Bacilli* Order: *Lactobacillales* Families: *Peptococcaceade*, *Desulfovibionaceae* Genera: *Clostridium* spp., *Staphylococcus* spp., *Clostridium* spp., *Enterococcus* spp., *Turicibacter* spp., Prevotella, Butyricicoccus
36.	Muchhala et al., 2022 [89]	Mouse	Morphine	(1) 75 mg pellet for 6 days SubQ (2) b.i.d. injections for 4 days i.p. (10 à 40 mg/kg)	No data	No data	No data
37.	Ren and Lotfipour, 2022 [46]	Rat	Fentanyl	IVSA of fentanyl at 0, 1.25, or 2.5 μg/kg/infusion during daily 2-h sessions for 5 days at a FR1 schedule of reinforcement, 2 days at FR2, followed by 2 days on FR5, all with a 20-s timeout	**Alpha diversity:** increased in controls after fentanyl IVSA use vs. before fentanyl IVSA **Beta diversity:** no data	In antibiotic-treated animals after vs. before fentanyl IVSA: Phylum: *Bacteroidetes*	-
38.	Ren and Lotfipour, 2022 [90]	Rat	Fentanyl	IVSA of fentanyl at 0, 1.25, or 2.5 μg/kg/infusion during daily 2-h sessions for 5 days at a FR1 schedule of reinforcement, 2 days at FR2, followed by 2 days on FR5, all with a 20-s timeout	**Alpha diversity:** increased after vs. before IVSA in males at 1.25 μg/kg/infusion decreased at Fentanyl IVSA at 1.25 vs. 0 μg/kg/infusion in females **Beta diversity:** fentanyl self-administration did not change beta-diversity	In males that self-administered fentanyl at 1.25 μg/kg/infusion: Phylum *Verrucomicrobia*; Genera *Ruminococcus* and *Akkermansia*	*Firmicutes*/*Bacteroidetes* ratios remained stable before and after fentanyl IVSA
						In females that self-administered 1.25 μg/kg/infusion: Genus *Prevotella*	
39.	H. Wang et al., 2022 [59]	Human	Oxycodone	Retrospective Cohort Study (Patients with Moderate to Severe Cancer Pain)	**Alpha diversity:** no significant changes Beta-diversity: no significant difference in gut microbiota diversity among the Control, Opioid-S, and Opioid-T groups	Genera: *Lactobacillus*, *Anaerostipes*, *Megamonas*, *Monoglobus*, and *Rikenellaceae_RC9_gut_group*	At the phylum level, there were no significant differences
40.	Ghosh et al., 2023 [53]	Mouse	Morphine	25 mg pellet for 1 or 2 days SubQ	**Alpha diversity:** decreased **Beta diversity:** altered composition and distinct clustering between study groups	Phyla: *Firmicutes* and *Verrucomicrobia* Families: *Enterococcaceae*, *Staphylococcaceae*, *Peptostreptococcoceae*, *Streptococcaceae*, *Erysipelotrichaceae*, *Pseudomonaceae*, *Akkermansiaceae*, *Coriobacteriaceae* Genera: *Staphylococcus*, *Enterococcus*, *Turicibacter*, and *Pseudomonas*	Phyla: *Bacteroidetes*, *actinobacteria*, and *tenericutes* Families: *Lactobacillaceae*, *Lachnospiraceae*, *Muribaculaceae*, *Ruminococcaceae*, *Burkholderiaceae*, *Eggerthelaceae*, and *Peptococcaceae* Genus: *Lactobacillus*
41.	Kolli et al., 2023 [42]	Mouse	Morphine	25 mg pellet SubQ à antibiotics for 7 days	**Alpha diversity:** increased **Beta diversity:** altered composition and distinct clustering between study groups	Species: *Parasutterella excrementihominis*, *Burkholderiales bacterium 1_1_47*, *Enterococcus faecalis*, *Staphylococus xylosus*, *Firmicutes bacterium M10–2*, *Bifidobacterium pseudolongum* and *Enterorhabdus caecimuris*	Species *Lactobacillus johnsonii*
42.	Truitt et al., 2023 [91]	Mouse	Morphine (withdrawal)	75 mg pellet for 3 days SubQ	During withdrawal: **Alpha diversity:** decreased **Beta diversity:** distinct clustering between study groups	At 2 h post-pellet removal: Phylum: *Verrucomicrobia*	At 2 h post-pellet removal: Phylum: *Firmicutes*
						-	At 12 h post-pellet removal: Phyla: *Verrucomicrobia*, *Firmicutes*, *Actinobacteria*
						-	At 24 h post-pellet removal: Phylum: *Actinobacteria*
43.	Abu et al., 2024 [31]	Mouse	Hydromorphone; methadone	Hydromorphone: 0.5 mg/kg, SubQ, b.i.d., pre-gestation day (PG) 1–3, 1.25 mg/kg, SubQ, b.i.d. PG 4–6, 2 mg/kg, SubQ, b.i.d. PG 7–9, 2.75 mg/kg, SubQ, b.i.d. PG 10–12, 3.5 mg/kg, SubQ, b.i.d. PG 13–14; transitioned to methadone (10 mg/kg, SubQ, b.i.d) until weaning of pups at 3 weeks of age	Mothers: **Alpha diversity:** no significant changes **Beta diversity:** altered composition and distinct clustering between study groups (methadone-treated vs. control dams)	Mothers: Genera: *Akkermansia* and *Bacteroides*; aerobic, biofilm forming bacteria, and gram-negative bacteria	Mothers: Genus *Turicibacter*; gram-positive bacteria
					Offspring: **Alpha diversity:** no significant changes **Beta diversity:** altered composition and distinct clustering between study groups (prenatally methadone-exposed and control offspring)	Offspring: Phyla: *Bacteroidota*, *Verrucomicrobiota*; Genera: *Akkermansia*, *Alistipes*, *Bacteroides*, *Butyricicoccus*, *Clostridium sensu stricto 1*, and *Lachnoclostridium* gram negative, aerobic, biofilm-forming, and gram-positive bacteria	Offspring: Firmicutes (phylum); Genera: *Lachnospiraceae A2*, *Anaeroplasma*, *Clostridium sp*. *ASF356*, *Bifidobacterium*, *Enterorhabdus*, *Erysipelatoclostridium*, *Family XIII UCG-001*, *Lachnospiraceae UCG-001*, and *Lactobacillus*
44.	Crawford et al., 2024 [35]	Mouse	Morphine	20 mg/kg once daily for 28 days SubQ	**Alpha diversity:** decreased **Beta diversity:** no data	-	SCFA-producing bacteria: Genus *Bacteroides*; Families: *Lachnospiraceae*, and *Ruminococcaceae*
45.	Greenberg et al., 2024 [37]	Rat	Heroin	IVSA: 20 μg/kg/100 μL infusion over 3 s. A session lasted for 12 h, or terminated once 300 infusions was reached. The IVSA phase lasted for 15 sessions	**Alpha diversity:** no significant changes **Beta diversity:** altered composition and distinct clustering between study groups (heroin vs. saline-yoked groups)	heroin self-administration phase: Genus *Bacteroides*; Families: *Lachnospiraceae*, *Muribaculaceae*	heroin self-administration phase: Genus *Alistipes*; Families: *Rikenellaceae*
						extinction phase: Genera: *Ruminoclostridium 6*, *Ruminiclostridium 5*; Family Muribaculaceae	extinction phase: Genera: *Mucisprillum*, *Ruminiclostridium 5*; Family *Lachnospiraceae*
46.	Hofford et al., 2024 [92]	Rat	Fentanyl	IVSA 2.5 μg/kg/infusion: daily 3-h sessions for 10 days at a FR1 schedule of reinforcement; then 2 days at FR2, 2 days at FR3, and 2 days on FR5 (increasing FR) or 6 days of FR1, then 2 d of PR and 2 days of FR1; then 20 d of home cage abstinence	**Alpha diversity:** no significant changes (opioids vs. saline) **Beta diversity:** no significant changes (opioids vs. saline)	-	Abundance of genera *Ruminococcus*, *Butyricicoccus*, *Lachnospiracae_unclassified*, and *Anaerotignum* negatively correlated with fentanyl intake during the last 2 d of fentanyl increasing FR or maintenance
47.	Inan et al., 2024 [61]	Rat	Oxycodone	Increasing doses for 12 days twice a day SubQ: days 1–4, 1 mg/kg; days 5–8, 2 mg/kg; days 9–12, 3 mg/kg	**Alpha diversity:** no significant changes **Beta diversity:** no data	-	-
48.	Kesh et al., 2024 [41]	Mouse	Morphine, oxycodone	Morphine for 5 weeks at escalating doses (10, 20, 30, 40, 50 mg/kg, i.p., b.i.d); Oxycodone for 5 weeks with at escalating doses of (5, 15, 25, 35, 45 mg/kg, i.p., b.i.d.)	**Alpha diversity:** increased in those from CP, Morphine, and CP + morphine mice with 11-week CP vs. controls No significant changes between mice treated with CP, Morphine, and CP + morphine **Beta diversity:** altered composition and distinct clustering between study groups (control mice vs. CP mice, morphine, or CP + morphine treatment groups; CP + morphine mice vs. CP-only mice)	CP Morphine vs. CP: Species: *Adlercreutzia caecimuris*, species from *Anaerotruncus*, *Adlercreutzia muris*	CP Morphine vs. CP: Species: *Lactobacillus johnsonii*, an *unidentified species from Lactobacillus*, and *Ducaniella muris*
					Similar **α-diversity** results were observed in oxycodone-treated CP animals in 11-week samples. Similar **β-diversity** results were observed in oxycodone-treated CP animals in 11-week samples.	CP Oxy vs. CP: Species: *Adlercreutzia muri*, unidentified species from *Enterorhabdus*, *Adlercreutzia mucosicola*	CP Oxy vs. CP: -

Abbreviations: SubQ—subcutaneous; i.p.—intraperitoneal; i.m.—intramuscular; b.i.d.—twice a day; d—days; h—hours; CLP—cecal ligation and puncture; IQR—interquartile range; HE—hepatic encephalopathy; SUD—substance use disorder; HC—healthy control; TLR2KO and TLR4KO—TLR2 and TLR4 Knockout; Ag—opioid agonist; AgAt—opioid agonist and antagonist At—opioid antagonist; At—opioid antagonist; N—neither opioid agonist nor antagonist; PO—prescription opioid; MMT—methadone maintenance therapy; DU—drug users; CD—compulsory detention; DSS—dextran sodium sulphate; MB—morphine baseline; MP—morphine post-treatment; SalineP—saline post-treatment; Mor-dep—morphine depressive; Mor-nondep—morphine non-depressive; CPP—conditioned place preference; POE—prenatal opioid exposure; F/B ratio—Firmicutes/Bacteroidetes ratio; POU—prescription opioid use; Opioid-T—opioid-tolerant group; Opioid-S—opioid-sensitive group; FR—fixed ratio; PR—progressive ratio; IVSA—intravenous self-administration; CP—chronic pancreatitis; Oxy—oxycodone.

### 3.2. The Impact of Opioid Use on Gut Barrier Function, Inflammation, and Short Chain Fatty Acid Levels

The reviewed studies suggest that opioid use, combined with gut dysbiosis, is linked to increased gut permeability, local and systemic inflammation, and reduced SCFA production.

#### 3.2.1. Opioid Use Increases Gut Permeability and Gastrointestinal Inflammation

**Figure 6:**
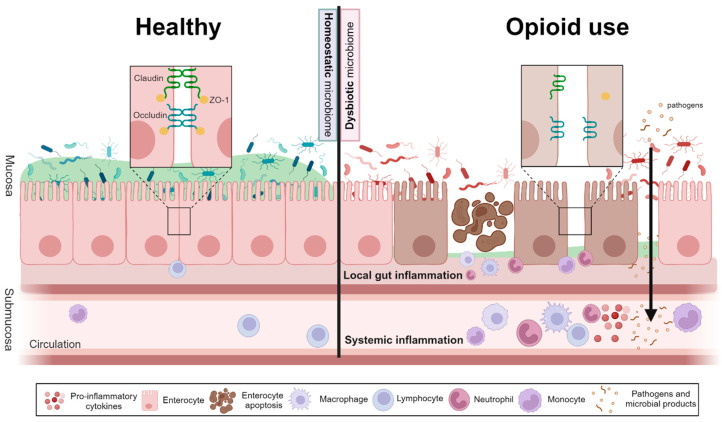
Impact of opioids on gut barrier function, permeability, and systemic inflammation. Current studies suggest that opioid use compromises gut barrier integrity by reducing the expression and altering the organization of tight junction proteins like Zonula occludens (ZO)-1 and Claudin. The disruption of these proteins allows pathogens and microbial products to translocate into the bloodstream. Additionally, opioids can potentiate a dysbiotic microbiome and promote gut inflammation, leading to further gut barrier compromise. This leads to activation of macrophages and results in increased recruitment of neutrophils and monocytes. Activation of these immune cells contributes to the upregulation of pro-inflammatory cytokines and increased enterocyte apoptosis. Figure was created with BioRender.com.

Experimental studies in mouse models have consistently shown that opioids disrupt gut barrier function and increase permeability, as indicated by reduced expression and altered organization of tight junction proteins ZO-1 and Claudin-1 [39,41,45,47], along with increased diffusion of fluorescein isothiocyanate (FITC)—dextran from gut to blood [54,67] (Figure 6). Bacterial translocation through the gut mucosa to other tissues was shown to be mediated by TLR2 and μ-opioid receptor signaling [17,18,64,65,67]. Notably, Jalodia et al. demonstrated that naltrexone treatment antagonizes morphine-induced disruption of the intestinal barrier [39], while Zhang et al. found that morphine-induced bacterial translocation was absent in germ-free or antibiotic-treated mice [65]. These findings suggest that both morphine and a dysbiotic microbiome contribute to the disruption of gut integrity.

Furthermore, opioids, particularly morphine, exacerbate gastrointestinal inflammation in mice, increasing epithelial cell apoptosis and upregulating pro-inflammatory cytokines like IL-6 [18,45,53]. Chronic morphine use in mice impairs epithelial integrity, increases inflammatory infiltrates in small intestinal villi, and causes significant histopathological changes in the gut of morphine-tolerant mice. Interestingly, fecal microbiota transplantation (FMT) from morphine-tolerant to germ-free or antibiotic-treated mice alone induces these histopathological changes, even without direct exposure to morphine [65]. In a model of dextran sodium sulphate (DSS)-induced colitis, hydromorphone treatment exacerbates DSS-induced damage, resulting in extensive mucosal damage, inflammatory cell infiltrates, and crypt architectural abnormalities [47]. Morphine treatment activates macrophages, recruits neutrophils, and increases the expression of chemokines associated with inflammation, contributing to a pro-inflammatory milieu and oxidative stress [39,42]. Additionally, morphine induces significant gastric inflammation, as evidenced by specific histological changes and increased expression of inflammatory cytokines in gastric tissue [53]. Another study reported that the gut inflammatory changes associated with a morphine-induced dysbiotic microbiome were attenuated in germ-free mice [42]. These findings collectively highlight the role of opioids in promoting gastrointestinal inflammation in murine models.

#### 3.2.2. Opioid Use Is Associated with Systemic Inflammation

In addition to local intestinal inflammation, opioid use has been associated with an increase in systemic inflammation.

**Figure 7:**
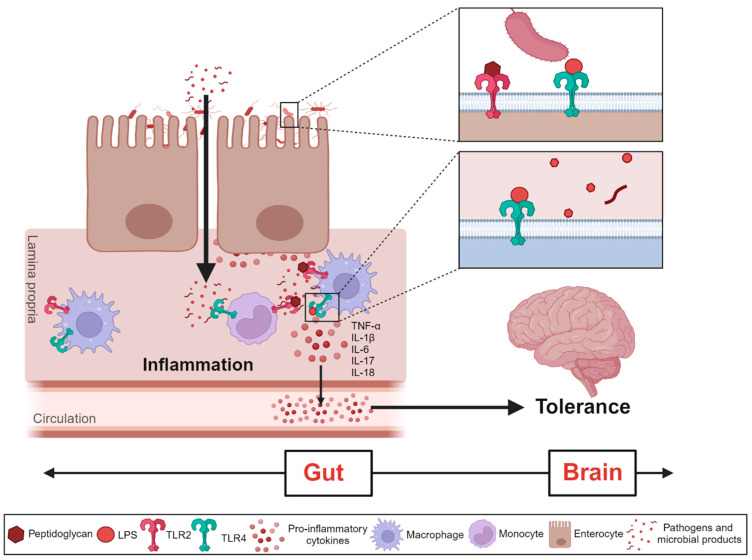
Association between opioid use and increased systemic inflammation, contributing to morphine tolerance through the gut-brain axis. Current studies suggest that opioids induce changes in gut microbiota, leading to intestinal bacterial product binding to enterocyte or gut immune cell TLR2 (recognizing bacterial peptidoglycan) and TLR4 (recognizing lipopolysaccharide (LPS)) receptors. This cascade leads to elevated levels and release of pro-inflammatory cytokines (such as TNF-α, IL-1β, IL-6, IL-17, and IL-18), leading to local gut inflammation and contributing to the development of morphine tolerance via the gut-brain axis. Figure was created with BioRender.com.

In murine models, opioid-induced dysbiosis activates TLR2/4 pathways, significantly increasing the release of proinflammatory cytokines such as TNF-α, IL-1β, IL-6, IL-17, and IL-18. Local intestinal inflammation contributes to the development of morphine tolerance via the gut-brain axis [17,18,31,47,65,67,89] (Figure 7).

The findings across multiple studies indicate a complex interplay between opioid use, gut dysbiosis, and inflammatory markers. For instance, Meng et al. found that in a mouse sepsis model, morphine treatment induced Gram-positive bacterial dissemination, up-regulating IL-17A and IL-6. Overexpression of IL-17A compromised intestinal barrier function, increasing bacterial dissemination and systemic inflammation. Notably, neutralizing IL-17A protected barrier integrity, reduced serum Il-6 levels, and improved survival in morphine-treated animals [64]. Similarly, Inan et al. demonstrated that blocking IL-17A not only prevented oxycodone-induced depression-like effects and hyperalgesia, but also reduced naloxone-precipitated withdrawal signs and normalized the increase in cytokine levels in the ventral tegmental area (VTA) [61]. Furthermore, comparative analysis of gene expression patterns across the morphine and antibiotic-treated groups revealed that mice with a depleted microbiome exhibited lower expression of inflammatory cytokines and chemokines, downregulation of the immune response, and reduced tissue damage following morphine treatment. Additionally, in germ-free mice, morphine did not upregulate TLR-4, proinflammatory cytokines (IL-6, IL-1β, IL-18, TNFα), chemokines (Cxcl1, Cxcl2, Cxcl17), or MMP16, unlike in mice with a morphine-induced dysbiotic microbiome. The study emphasizes the role of a dysbiotic microbiome as a mediator in inflammation induced by morphine [42].

Human studies have also reported an increase in pro-inflammatory cytokines in plasma in relation to opioid use and altered gut microbiota. Acharya et al. found that opioid-using cirrhotic patients exhibited distinct alterations in the microbiome alongside elevated levels of endotoxin and IL-6 compared to non-opioid users. However, IL-17 levels were similar between the two groups [63]. In a retrospective study by Wang et al., patients with moderate to severe cancer pain who were taking oxycodone exhibited elevated levels of IL-2, IL-4, IL-6, IL-10, TNF-α, and IFN-γ compared to the patients without cancer pain [59]. Cruz-Lebrón et al. observed elevated plasma IL-6 and TNFα levels in methadone-treated patients compared to non-opioid users. They also found negative correlations between immune mediators and plasma SCFAs, as well as fecal bacterial abundance. Specifically, MIP1α negatively correlated with plasma acetate and butyrate, while lipocalin 2 negatively correlated with the relative abundance of *Bacteroidetes* and *Verrucomicrobia*. These results suggest that immune mediator levels vary with bacterial abundance and SCFA production, highlighting the impact of chronic opioid use on the balance between the gut microbiome and immune mediators [56].

These findings underscore the intricate relationship between opioid use, gut microbiota dysbiosis, and systemic inflammation. Further research investigating the mechanistic pathways underlying these interactions and their clinical implications could provide valuable insights into novel therapeutic interventions aimed at addressing the complex challenges associated with opioid use and gut microbiota dysregulation.

#### 3.2.3. Opioid Use Results in Reduced Short Chain Fatty Acid Levels

SCFAs, like acetate, propionate, and butyrate, are synthesized in the colon through bacterial fermentation of dietary fibers and resistant starch. They play crucial roles in gastrointestinal physiology, immune function, host metabolism, and CNS development and homeostasis, exerting local and systemic effects. SCFAs contribute to the gut membrane integrity, anti-inflammatory responses, alteration of chemotaxis, and phagocytosis. SCFAs may directly influence the brain by reinforcing blood-brain barrier integrity, modulating neurotransmission, inhibiting microglia activation, influencing levels of neurotrophic factors, and promoting memory consolidation [93,94,95,96,97].

In addition to noting a reduction in SCFA-producing bacteria, several reviewed studies suggest that opioid use directly affects SCFA production, as evidenced by decreased levels observed in fecal and plasma samples [35,56]. For instance, Cruz-Lebrón et al. found that patients undergoing methadone maintenance treatment displayed significantly lower fecal acetate, propionate, and butyrate levels compared to non-opioid users [56]. Similarly, in a mouse study, Crawford et al. demonstrated that chronic morphine treatment reduced fecal levels of butyrate and propionate but not acetate. However, these reductions were reversed by interventions like ketogenic diet, fecal microbiota transplantation (FMT) with feces from ketogenic diet–treated mice, or rectal administration of SCFA-producing bacteria. Moreover, supplementing with SCFAs, including butyrate, delayed the onset of opioid-induced hyperalgesia (OIH), suggesting a direct role for SCFAs in mitigating opioid-related adverse effects [35].

In summary, the evidence suggests a significant interplay between opioid use, SCFAs, and gut microbiota, highlighting the potential therapeutic implications of targeting SCFA pathways to mitigate adverse effects associated with opioid therapy.

## 4. Gut Microbiota Impact on Opioid Use Disorder

Emerging research indicates that the gut microbiome is not only altered by opioid use but also plays a role in modulating opioid use and potentially influencing the development of opioid use disorder. Additionally, the gut microbiome has been shown to influence the behavioral reactions of rodents to other psychoactive substances, such as cocaine and methamphetamine [43,98,99]. Below, we will summarize findings from various studies that have explored how alterations in gut microbiota can influence opioid use through the development of inflammation, changes in the brain, altered behavior, and tolerance development.

### 4.1. Associations between Gut Microbiota and Antinociceptive Tolerance to Opioids

Analgesic tolerance is a progressive reduction in pain relief during chronic opioid use, necessitating dose escalation to maintain initial effects and increasing the risk of addiction and fatal overdose [100]. Research has focused on developing opioid analgesic tolerance through opioid receptor desensitization, receptor downregulation, intracellular signaling changes, and neuroadaptations [101,102]. Recently, various experimental approaches were used to assess the role of gut microbiome and opioid-induced dysbiosis in antinociceptive tolerance.

Evidence from preclinical studies suggests an association between gut microbiota, inflammation, and antinociceptive tolerance to opioids. It has been reported that depleting gut bacteria with antibiotics prevents morphine-induced increases in gut permeability, mucosal destruction, local inflammation, and antinociceptive tolerance in mice [42,67]. Similarly, Zhang et al. reported that morphine analgesic tolerance was significantly attenuated in GF and pan-antibiotic-treated mice, and reconstitution of GF mice with naïve fecal microbiota reinstated morphine analgesic tolerance [65]. Wang et al. demonstrated that morphine use increased pathogenic bacterial communities, and *E. faecalis* augmented morphine analgesic tolerance in mice [50]. The presence of induced colitis in mice led to greater antinociceptive tolerance to chronic morphine exposure compared to mice without colonic inflammation, suggesting a peripheral component to opioid tolerance development [88]. Using colonic supernatants, Mischel et al. have shown that gut mediators from morphine-exposed mice induce tolerance in naive dorsal root ganglion neurons. Oral vancomycin mitigated this cellular level tolerance in primary afferent neurons [86]. Besides, in a retrospective human study, H. Wang et al. suggested that analgesic tolerance induced by long-term oxycodone use may be closely linked to systemic inflammation, as indicated by the consistent upregulation of plasma IL-6 and TNF-α levels, alongside a significant increase in leukocytes and neutrophils and a substantial decrease in lymphocytes [59]. Overall, these findings establish the dysbiotic microbiome as a mediator of opioid-induced gut pathophysiology, systemic inflammation, and antinociceptive tolerance to opioids.

### 4.2. Gut Microbiome Induces Changes in the Brain during Different Stages of Opioid Use

Evaluating the neurobiology of addiction development reveals three distinct stages: binge/intoxication, withdrawal/negative affect, and preoccupation/anticipation. Each stage is associated with specific brain areas, particularly the nucleus accumbens (NAc), amygdala, and prefrontal cortex [1,103,104]. Prolonged opioid consumption has been shown to induce neuroinflammation in the central nervous system, evidenced by the increased release of proinflammatory cytokines (e.g., TNF-α, IL-1β, and IL-6) by glial cells, contributing to the development of opioid tolerance, hyperalgesia, and dependence [105,106,107,108,109]. Recent experimental studies have explored how the gut microbiota, in association with opioid use, impacts neuroinflammation, alters brain function, and influences withdrawal and abstinence.

Several studies have documented neuroinflammatory changes associated with alterations in gut microbiota and opioid use. Lee et al. discovered that intermittent morphine treatment triggered microglial activation, hyperalgesia, and impaired reward response, while sustained treatment did not. Surprisingly, depleting the gut microbiota with antibiotics replicated neuroinflammation and its effects, such as reduced opioid analgesic potency and impaired cocaine reward after intermittent morphine treatment. Reintroducing a control microbiota reversed the microglial activation state and behaviors [43]. O’Sullivan et al. observed upregulation of neuroinflammatory genes, particularly *Tnf*, heightened astrocyte activity, and a decreased Firmicutes to Bacteroides ratio, indicating gut dysbiosis during opioid withdrawal. The elevated *Tnf* expression suggests that local paracrine signaling in the central nucleus of the amygdala (CeA) during opioid withdrawal shifts toward a neuroinflammatory state, as confirmed by increased TNF-α protein levels. They propose that these inflammatory and gut microflora changes contribute to the negative emotions experienced during opioid withdrawal, thereby driving dependence [68].

Furthermore, other authors have documented specific transcriptional and functional changes in the brain associated with altered gut microbiota. Hofford et al. found that microbiome depletion in morphine-treated mice induces substantial gene expression changes in the NAc, a structure implicated in substance reward responses [38]. They also conducted a global proteomic analysis of the NAc following microbiome manipulation and fentanyl administration to elucidate how microbiome status influences the functional proteomic landscape in this key limbic substructure. Their findings demonstrate that microbiome depletion leads to significant changes in the synaptic proteome in response to repeated fentanyl treatment, potentially enhancing motivation for drug administration [92]. Simpson et al. showed that oxycodone administration increased the recruitment of Fos-positive (Fos+) neurons, a marker of neuronal activation, in the basolateral amygdala during intoxication, while withdrawal led to increased recruitment of Fos+ neurons in the periaqueductal gray, central nucleus of the amygdala (CeA), locus coeruleus, paraventricular nucleus of the thalamus, agranular insular cortex, bed nucleus of the stria terminalis, and lateral habenula medial parvocellular region. Gut microbiome depletion altered neuronal activation patterns during both oxycodone intoxication and withdrawal, affecting brain regions implicated in opioid dependence. Furthermore, microbiome depletion disrupted functional connectivity among these brain regions during both states, suggesting an important modulatory role for the gut-brain axis in the neural mechanisms underlying opioid use and dependence [48].

Further studies have explored the impact of gut microbiota on opioid withdrawal and abstinence. In a murine model of opioid dependence, mice receiving FMT from morphine-treated donor mice showed fewer naloxone-precipitated jumps compared to those receiving FMT from saline-treated donors. Additionally, a regimen of broad-spectrum antibiotics mitigated naloxone-precipitated morphine withdrawal in morphine-dependent mice [54]. Similarly, Truitt et al. found that germ-free mice lacking the microbiome did not develop somatic morphine withdrawal symptoms. Their study also revealed that antibiotic treatment altered withdrawal timing and duration [91]. Another study, conducted by Ji et al., investigated the effects of morphine abstinence on the gut microbiota and its association with depressive behavior. They found that four weeks of morphine abstinence altered the gut microbiota’s composition, but not its richness. Specific microbial taxa were identified as markers for depressive and non-depressive groups. The findings suggest a potential link between gut microbiota dysbiosis and morphine abstinence-induced depressive behavior [55].

Overall, these findings underscore the significant role of the gut microbiome in the development of specific changes in the brain during different stages of opioid use and dependence.

### 4.3. Changes in Microbiome Alter Behavioral Response to Opioids

The studies exploring the connection between the gut microbiota and opioid use consistently demonstrate that changes in gut microbiota can modulate behavioral responses to opioids.

Zhang et al. provided direct evidence that morphine alters gut microbiota in rats, linking these changes to sensitivity to morphine reward. Using the conditioned place preference (CPP) paradigm, they categorized morphine-treated rats into low and high CPP (L- and H-CPP, respectively) groups based on their CPP scores. Interestingly, these groups exhibited distinct microbial compositions before and after morphine treatment, with the relative abundance of specific taxa correlating with the CPP score at baseline and after morphine treatment. Significant baseline differences in genera *Olsenella*, *Rothia*, and *Helicobacter* were noted between the groups, with *Rothia* negatively correlated with CPP scores. The authors suggested that decreased *Olsenella* and *Rothia*, along with increased *Helicobacter* at baseline, may predict a higher risk of addictive behaviors with morphine exposure [51].

Investigating the dynamics of gut microbiome changes during various phases of morphine-induced CPP, Zhang et al. observed an increase in Verrucomicrobia abundance during repeated morphine conditioning, followed by a decrease during the extinction stage. Conversely, the abundance of Bacteroides exhibited an opposite trend. Particular taxa that best characterize each morphine-CPP stage were determined, serving as distinctive microbiota biomarkers. Metabolic pathways related to amino acids showed differing levels of activity or prevalence at different stages of CPP [52].

Ren and Lotfipour found that gut microbiome depletion increased fentanyl self-administration in male rats, particularly at lower reinforcement schedules, but not in females. However, both genders showed higher fentanyl intake at higher reinforcement schedules compared to water drinking controls. Replenishing microbial metabolites via SCFA administration reduced fentanyl self-administration [46]. Their subsequent study revealed that bacterial diversity predicts responses to fentanyl infusions at a progressive ratio schedule of reinforcement in a sex- and dose-dependent manner [90].

Hofford et al. observed a decrease in morphine CPP in mice after microbiome knockdown with nonabsorbable antibiotics, particularly evident at higher doses. In a separate study using a self-administration and drug-seeking model, they found that microbiome depletion amplified motivation for fentanyl intake. This heightened motivation showed a negative correlation with the abundance of specific bacterial genera, such as *Ruminococcus*, *Butyricicoccus*, *Lachnospiraceae_unclassified*, and *Anaerotignum*, suggesting their potential role in fentanyl’s reinforcing properties [38,92].

In summary, these findings collectively underscore the critical relationship between gut microbiota, its metabolites, and behavioral responses to opioids, further suggesting a significant implication for the involvement of the microbiota in the development of OUD.

## 5. Gut Microbiota Modulation as a Treatment Option for Opioid Use Disorder

Considering the intricate pathophysiological relationship between gut microbiota and brain alterations at various stages of addiction, experimental studies have explored potential therapeutic measures to modulate the gut microbiome and its impact on opioid responses.

### 5.1. Short-Chain Fatty Acids (SCFAs) for Treatment of Opioid Use Disorder

As previously discussed in this review, opioids induce gut dysbiosis and reduce levels of SCFAs. Therefore, experimental models have investigated the potential benefits of SCFAs in mitigating opioid-related effects [35,38,45,46,55,92]. Several experimental studies in rats have reported that microbiome depletion increases fentanyl self-administration and drug seeking behavior after abstinence. Additionally, replenishing SCFAs after antibiotic treatment reverses fentanyl self-administration levels to those similar to control animals [46,92]. In an earlier study, Hofford et al. observed that depleting the gut microbiome with antibiotics in male mice exposed to high-dose morphine resulted in reduced morphine-induced CPP and locomotor sensitization, along with significant changes in gene expression within the NAc [38]. Replacing SCFA metabolites, which were diminished by microbiome knockdown, reversed both the behavioral and transcriptional effects observed. Overall, these findings show the potential role of SCFA in regulating the behavioral response to opioids.

Crawford et al. demonstrated in a mouse study that a ketogenic diet or SCFA supplementation delayed or alleviated opioid-induced hyperalgesia [35]. SCFAs have also been implicated in regulating gut microbiota and behavior associated with methamphetamine and cocaine use [98,99,110].

In summary, SCFAs play a significant role in microbiome-brain communication and show promise in mitigating adverse opioid effects in experimental studies. Further clinical research is needed to validate these findings and develop effective interventions for improving opioid treatment outcomes.

### 5.2. Probiotics for Treatment of Opioid Use Disorder

Several experimental studies have also explored the use of probiotics to reduce the adverse effects of opioids. Zhang et al. demonstrated that pretreatment with VSL#3 significantly reduced morphine antinociceptive tolerance compared to sham mice. This pretreatment also partially restored gut microbial components, reduced immune cell infiltration, and mitigated morphine-induced increases in TLR expression and proinflammatory cytokine levels [65]. Abu et al. found that prenatal exposure to methadone in mice resulted in alterations in gut microbiota composition, elevated inflammatory markers, and heightened sensitivity to thermal and mechanical pain in offspring. Supplementing the probiotic VSL#3 in dams alleviated this hypersensitivity in prenatally methadone-exposed offspring. Additionally, modulation of the maternal and neonatal gut microbiome with probiotics induced transcriptional changes in genes associated with neuropathic and immune-related signaling, observed in both whole brain and midbrain samples of the offspring [31]. However, Thomaz et al. reported no effect of pretreatment with probiotics (*Bifidobacterium longum* or *Lactobacillus rhamnosus*) on naloxone-precipitated withdrawal in morphine-dependent mice [54].

In conclusion, while these studies offer insights into the potential of probiotics in influencing opioid-related outcomes, the mixed results underscore the complexity of the gut microbiome’s role in modulating such responses, highlighting the necessity for further research in this area.

### 5.3. Fecal Microbiota Transplantation for Treatment of Opioid Use Disorder

FMT offers a direct method to alter the gut microbiota and potentially provide therapeutic benefits. While clinically indicated for refractory *Clostridium difficile* infection, ongoing research is exploring its applications in various gastrointestinal and extra-gastrointestinal diseases. Experimental animal models have also investigated FMT’s role in relation to opioid use. Banerjee et al. demonstrated that transplanting placebo-treated microbiota into morphine-treated animals rescued morphine-induced microbial dysbiosis and gut barrier disruption [17,111]. Zhang et al. showed that morphine analgesic tolerance was significantly attenuated in germfree and pan-antibiotic-treated mice. Reconstitution of germfree mice with naïve fecal microbiota reinstated morphine analgesic tolerance [65]. Lee et al. revealed that intermittent morphine treatment triggered microglial activation, hyperalgesia, and impaired reward response. Surprisingly, depleting the gut microbiota via antibiotic treatment mirrored neuroinflammation and its consequences, including reduced opioid analgesic potency and impaired cocaine reward. However, colonizing antibiotic-treated mice with a control microbiota restored microglial activation and behaviors [43]. Thomaz et al. found that morphine-dependent mice receiving FMT from morphine-treated donors exhibited fewer naloxone-precipitated jumps, indicating less somatic signs of withdrawal compared to those receiving FMT from saline-treated donors. Although morphine treatment altered microbial contents in the mouse cecum, they were not differentially impacted by FMT [54].

Overall, these studies collectively highlight the significant role of the gut microbiota in modulating opioid-related outcomes.

## 6. Discussion

In this comprehensive review, we summarize the current evidence on the bidirectional association between opioid use and gut microbiota and its role in the development of OUD. This relationship reveals a complex interplay that profoundly affects both gut and brain health. Evidence from animal and human studies suggests that opioids induce gut dysbiosis, characterized by changes in bacterial diversity [17,30,31,32,34,35,36,37,38,39,40,41,42,43,45,47,48,49,50,51,52,56,57,58,59,60,63,64,65,90], disrupted gut microbial composition [17,30,31,32,34,35,36,37,38,39,40,41,42,43,45,46,47,48,49,50,51,52,56,57,58,59,60,63,64,65,66,67,68], and reduced SCFA levels [35,56]. Some studies showed an increase in potentially pathogenic bacteria [17,37,39,42,43,45,47,50,53,58,64,66], while others reported a decrease in beneficial SCFA-producing commensals [17,31,35,36,37,39,40,41,42,43,45,47,53,56,57,63,65]. This implies that therapeutic interventions could focus on managing pathogenic bacteria or increasing beneficial ones. Moreover, long-acting μ opioid receptor agonists used for OUD maintenance treatment, such as methadone and buprenorphine, appeared to be associated with dysbiosis, highlighting the potential for microbiome manipulations as adjunct therapy to improve treatment success [56,57]. Moreover, several preclinical studies have identified specific gut microbiome taxa as biomarkers for predicting increased risk of opioid addictive behaviors [51] or a higher risk of opioid abstinence-induced depression [55]. This underscores the potential of bacterial population characterization as a source of biomarkers for OUD treatment efficacy. However, the particular changes in microbiome diversity with opioid use appeared variable. They ranged from decreased alpha diversity [34,35,40,45,47,50,56,57] to no changes [17,37,38,43,48,49,51,54,55,61] or even an increase in alpha diversity [36,39,41,52]. Moreover, a recent Mendelian randomization study did not find a causal relationship between prescription opioid use and changes in the gut microbiome [85]. This highlights the need for longitudinal clinical studies to evaluate the impact of opioids on gut microbiome.

Analyzing the pathogenetic mechanisms, opioid-induced dysbiosis was linked to compromised gut permeability [17,18,31,39,41,45,47,54,64,65,67] and heightened local and systemic inflammatory response [17,31,42,45,47,56,59,61,63,64,65,67,89,112]. These alterations contribute to a cascade of physiological and behavioral effects that exacerbate opioid use and dependence [38,42,43,46,48,50,51,52,54,55,59,65,67,68,88,90,91,92]. In particular, experimental studies highlight the significant influence of the gut microbiota on opioid use through mechanisms involving altered gene expression in the NAc [38,92], modifications in reward and addiction pathways [48], and shifts in pain sensitivity [50,59,65,88]. These findings emphasize the direct link between the gut microbiota and substance-triggered reward responses [38,46,51,52,90,92] and withdrawal states [54,68,91], underscoring its significant role throughout various stages of addiction. This intriguing physiological context suggests that altering the gut microbiome might have the potential to mitigate the adverse effects of opioid use and improve adherence to opioid agonist therapy for treating OUD.

In preclinical studies, SCFA supplementation has shown potential in mitigating opioid-induced hyperalgesia and reducing drug-seeking behaviors by restoring microbial balance [35,38,45,46,55,92]. Probiotics, particularly formulations like VSL#3, have exhibited efficacy in reducing opioid tolerance and inflammatory responses, although results are mixed and further research is needed [31,54,65]. FMT has also been shown to reverse opioid-induced dysbiosis and its associated adverse effects [17,43,54,65]. Moreover, dietary modifications, including ketogenic diets [35] and omega-3 polyunsaturated fatty acids [87], have shown varying degrees of success in preclinical models, suggesting their potential to improve treatment outcomes for opioid-related disorders. These findings collectively highlight the critical role of the gut-brain axis in the pathogenesis of opioid addiction and underscore the potential of microbiome-targeted therapies to alleviate opioid-related harm. Future clinical research is essential to validate these experimental insights and develop effective, microbiome-based interventions to improve the health and recovery of individuals with opioid use disorder, including longitudinal studies examining gut microbiome changes during OAT and their correlation with treatment outcomes.
